# Impacts of different triathlon races on systemic cytokine profile and metabolic parameters in healthy individuals: a systematic review

**DOI:** 10.1186/s13102-023-00763-8

**Published:** 2023-11-06

**Authors:** Matheus Santos de Sousa Fernandes, Jefferson Mariano Gomes, Felipe J. Aidar, Mabliny Thuany, Tayrine Ordonio Filgueira, Raphael Fabrício de Souza, Georgian Badicu, Fatma Hilal Yagin, Gianpiero Greco, Stefania Cataldi, Angela Castoldi, Abdullah F. Alghannam, Fabrício Oliveira Souto

**Affiliations:** 1 Programa de Pós-graduação em Neuropsiquiatria e Ciências do Comportamento, Centro de Ciências da Médicas, Universidade Federal de Pernambuco, Recife, Pernambuco Brazil; 2 Instituto de Imunopatologia Keizo Asami, Universidade Federal de Pernambuco, Recife, Pernambuco Brasil; 3 Faculdade de Comunicação Turismo e Tecnologia de Olinda, Olinda, Pernambuco, Brasil; 4 Department of Physical Education, Federal University of Sergipe, São Cristovão, Sergipe, Brazil; 5 Center of Research, Education, Innovation and Intervention in Sport (CIFI2D), Faculty of Sport, University of Porto, Porto, Portugal; 6 Department of Physical Education and Special Motricity, Faculty of Physical Education and Mountain Sports, Transilvania University of Braşov, Braşov, 500068 Romania; 7 Department of Biostatistics and Medical Informatics, Faculty of Medicine, Inonu University, Malatya, 44280 Turkey; 8 Department of Translational Biomedicine and Neuroscience (DiBraiN), University of Study of Bari, Bari, 70124 Italy; 9 Lifestyle and Health Research Center, Health Sciences Research Center, Princess Nourah bint Abdulrahman University, Riyadh, Saudi Arabia

**Keywords:** Physical training, Cytokines, Chemokines, Biomarkers, Performance

## Abstract

The present systematic review aimed to discuss the impacts of different triathlon protocols on the level of pro and anti-inflammatory cytokines, as well as biomarkers related to the performance of healthy individuals. Four databases [PubMed (28 articles), Scopus (24 articles), Science Direct (200 articles), and SPORT Discus (1101 articles) were assessed. The eligibility criteria were applied, and the selected articles were used in the peer review, independently, as they were identified by March 2022. Of the 1359 articles found, 10 were included in this systematic review. Despite the difference in triathlon protocols, it was observed an increase in pro and anti-inflammatory cytokines including IL-4 and IL-10, and chemokines, such as IL-8 and MCP-1. Moreover, the anti-inflammatory serum levels increase after triathlon. Overall, the studies also reported enhancement in the serum levels of cortisol, creatine kinase, C reactive protein, Endothelial Growth Factor, Vascular Endothelial Growth Factor, Myostatin, Lactate dehydrogenase, free fatty acids, and lactate dehydrogenase in triathlon athletes. This systematic review indicates that different triathlon race promotes an acute elevation of circulating cytokines and chemokines levels which return to standard levels after triathlon races. The findings of this systematic review demonstrate that the modulation of inflammatory parameters may be associated with an increase in metabolic indicators (CK, Cortisol, and LDH) after the end of different types of triathlon races.

## Introduction

Triathlon is a multisport discipline, composed of swimming, cycling, and running, performed sequentially and in different environmental contexts [1]. The Triathlon reached the Olympic level at the Sydney Games (2000) and, today, it has six officially recognized distances: (1) Sprint (750 m of swimming, 20 km of cycling, and 5 km of racing); (2) Olympic, (1.5 km swimming, 40 km cycling, and 10 km running); (3) Long Distance (with distances covered that add up to three times the Olympic distance); (4) Mixed Relay, (300 m swim, 8 km bike, and 2 km run) and the routes of (5) Ironman (3.8 km swim, 180 km bike, and 42 km run), and (6) Half-Ironman (half of these routes) [2, 3]. With the rise of the number of race events and triathletes over the last years, the research interest is to enhance performance and prevent injuries arising from constant exposure of athletes to large volumes of physical effort [4, 5].

Evidence shows that triathlon events have a well-defined biochemical and physiological characterization according to the profile of intensity (light to moderate) and duration (long), which impacts on the recruitment of the oxidative pathway, made up of various reactions and energy mechanisms, including aerobic glycolysis, the Krebs cycle, electron transport chain and mitochondrial beta-oxidation, promoting sufficient energy input for your motor actions during the triathlon races [2, 6–9].

The training and competition load associated with this sports practice, promote acute and chronic physiological changes, mainly related to cardiorespiratory fitness determinants, such as maximal oxygen volume, anaerobic threshold, and running economy. Running economy is defined as the energy cost of running, that is, the volume of oxygen consumed per distance covered, which is reported to promote changes in the body composition, including better control of body weight [10, 11]. Considering the training load to which the athletes are exposed, the evidence available demonstrates the importance of applying biological analysis, including metabolic and immunological markers (cytokines and cellular activity), aiming to improve sports performance [12–14], and reducing injury risk.

Multiple factors, including modalities of physical exercise with variability of intensity of physical exertion, are reported to affect cytokine [15]. For instance, Filgueiras et al. demonstrate that maintaining high levels of activity and physical fitness can promote positive immunomodulation in components of innate and acquired immunity [16]. In addition, chronically the practice of physical exercise at moderate to vigorous intensity can increase the production of anti-inflammatory cytokines such as interleukins IL-6, IL-10, and IL-1a receptor antagonist (IL-1ra). Thus, avoiding immunosuppression and establishment of opportunistic infections [7, 17].

In this sense, when exposed to high demands of physical effort for long periods associated with high volume and/or intensity demands, athletes may present a decrease in the immune response against different pathogens including viruses and bacteria, and the establishment of infections including in the upper respiratory tract, mainly [18, 19]. However, the real effects of races such as triathlon in the production of cytokines and adjunct compounds is still not very clear and it is extremely necessary. Therefore, the aim of the present systematic review is to analyze the impacts of different triathlon races on the levels of pro and anti-inflammatory cytokines, as well as metabolic markers related to the performance of healthy individuals.

## Methods

The present systematic review was performed following the Preferred Reporting Items for Systematic Review and Meta-Analysis (PRISMA) [20].

### Database and search strategy

The search strategy was led by MSSF in collaboration with TOF. Following, we conducted a systematic search in the PubMed, Scopus, SPORT Discus, and Science Direct databases of studies published until March/2022. The search terms were considered appropriate based on the Medical Subject Headings database (*MeSH* terms). In the PubMed (*Medline*), Scopus, Science Direct, and SPORT Discus the search strategy was used as search terms: (((Triathlon) OR (Triathlon Training)) OR (Iron Man Triathlon)) AND ((Cytokine) OR (Cytokines)). The selected studies describe the possible impacts of triathlon on cytokine expression and metabolic markers associated with performance in in triathlon athletes.

### Selection of articles

In the first step, two authors (TOF and MSSF) independently assessed the titles and abstracts of each article found. Then, for abstracts that contained information according to the inclusion and exclusion criteria, the full text was read to observe the presence or absence of eligibility criteria. Duplicates were removed by creating an EndNote library, version 20. Possible discrepancies between evaluators were resolved by consensus. Studies that met the eligibility criteria for **PICOS** criteria were included in the study **(**Table 1**).** Articles were excluded if do not present a triathlon group, inclusion of samples with associated diseases or pharmacological strategy, reviews, opinions, letters for editor, comentar, animal studies, or full text unavailable.

**Table 1:** PICOS strategy for studies eligibility

Strategy	Inclusion criteria	Exclusion criteria
Population	Triathletes	Non Triathletes
Intervention	Triathlon	Any other type of physical activity, exercise or sport
Comparator	Individuals who did not perform triathlon intervention	Individuals exposed to pharmacological interventions, with associated pathological or psychiatric conditions
Outcomes	Cytokines: Pro and anti-inflammatory Metabolic markers Creatine Kinase (CK) Free Fatty Acids C-Reactive Protein (CRP) Endothelial Growth Factor (EGF) Vascular Endothelial Growth Factor (VEGF) Cortisol Myostatin Lactate dehydrogenase (LDH)	Any parameter not related to pro- and anti-inflammatory cytokines and metabolic parameters linked to physical effort through triathlon practice
Study design	Intervention studies	Animal’s studies, review’s, letter for editor, comments, case report.

### Data extraction and methodological Quality Assessment

Data were extracted by two independent researchers (MSSF e JMG) using an Excel spreadsheet and considering: (1) Author and year; (2) Population (age, sex, number of participants, country); (3) Intervention (type and duration), and (4) Pro and anti-inflammatory cytokines and metabolic markers related with performance. Discrepancies were evaluated by a third evaluator (GCJS).

The “Joanna Briggs Institute (JBI) Critical Appraisal Checklist for Analytical Randomized Controlled Trial and Non-Randomized Experimental Studies” was used to verify the methodological quality of the included articles (Reference). This tool consists of eight questions that assess the methodological quality of articles based on the following criteria: (1) Was the inclusion criteria well defined?; (2) Have participants and context been described in detail?; (3) Were the measurements collected in a valid and reliable way?; (4) Were standardized and objective inclusion criteria used?; (5) Were any confounding variables found?; (6) Were strategies used to deal with confounding variables?; (7) Were the results measured validly and reliably?; and (8) Was the statistical analysis used adequate? The questions were answered with “Yes”, “No” or “Unclear”. When the answer was “yes”, a score was given, when the answer was “no” or “undefined”, no score was given. The score for each article was calculated as a percentage and the quality of each study was classified as high (80–100%), fair (50–79%), or low (50%). All studies were independently reviewed by two reviewers (TOF and MSSF). Discrepancies between raters were resolved by consensus [21].

## Results

### Characterization of included studies

**Fig. 1:**
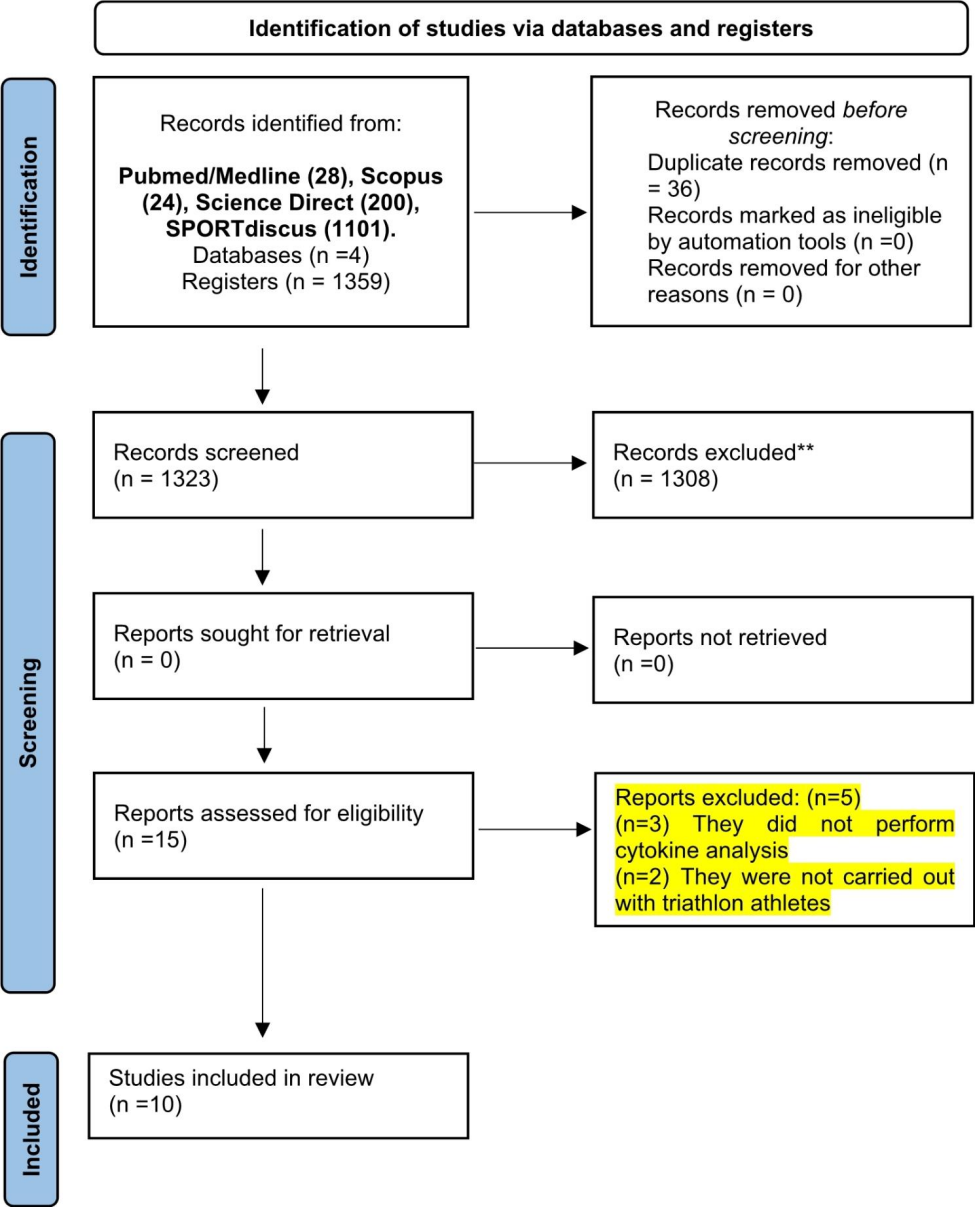
PRISMA 2020 flow diagram for new systematic reviews which included searches of databases and registers only; *Consider, if feasible to do so, reporting the number of records identified from each database or register searched (rather than the total number across all databases/registers); **If automation tools were used, indicate how many records were excluded by a human and how many were excluded by automation tools

A total of 1359 studies were selected from the following databases: PubMed/Medline (28); Scopus (24); Science Direct (200); SPORT Discus (1101). A total of 36 duplicated studies were deleted via Endnote software. Then, titles and abstracts of 1323 articles were read, and 1308 were excluded, as they did not meet the eligibility criteria. A total of 15 articles were read in full, 5 of which were excluded as it does not fully comply with the purpose of this review. Finally, 10 studies were included in this systematic review (Fig. 1).

The studies were published between 2000 and 2019. Seven studies [22–28] evaluated only male subjects and three studies [29–31] included both sexes. Among the nationalities within the included studies, two studies were carried out in Brazil [23, 27]; USA [30, 31]; and France [24, 29]; one study in Austria [26]; Australia [28]; Italy and China [25]. The average age of the participants was 33.5 ± 0.70 years. Different triathlon races were used in the studies included in this review: Iron Man [26–29]; Long-Distance [24]; Sprint Triathlon [25]; Top-level Triathlon [22]; Olympic Triathlon [23]; Ultraman Triathlon [31]; and World Championship [30]. The races had a Swim average of 7 ± 4.2 Km; Cycle 180 ± 68.7 Km and Run 20.0 ± 17.2 Km, (Table 2**).**

**Table 2:** Characteristics of the Studies included

Author, year	*n*	Gender	Country	Age Group (Mean ± SD)	Triathlon Race Type	Swim (km)	Cycle (km)		Run (km)	
Banfi, 2008	07	Male	Italy	31.5 ± 1.5	Top-level Triathlon					
Bassit, 2000	12	Male	Brazil	25.5 ± 3.2	Olympic Triathlon	1.5		40		10
Huang, 2019	18	Male	China	21.1 ± 1.5	Sprint Triathlon	0.75		20	5	
Jenkendrup, 2008	30	Male/Female	France	33.0 ± 6.0	Ironman Triathlon	3.8		185	42.2	
Merino, 2006	12	Male	France	34.8 ± 1.4	Long-Distance	4.0		120	30	
Neubauer, 2008	42	Male	Austria	35.3 ± 7.0	Ironman Triathlon	3.8		180	42.2	
Nieman, 2004	38	Male/Female	EUA	35.2 ± 1.6	World Championship	3.9		180	42	
Pinho, 2010	18	Male	Brazil	34.7 ± 2.15	Ironman Triathlon	3.8		180	42.2	
Smith, 2019	18	Male/Female	EUA	37.4 ± 7.7	Ultraman Triathlon	10		144.8	84.4	
Suzuki, 2006	09	Male	Australia	34.0 ± 5.0	Ironman Triathlon	3.8		180	42.2	

**Caption: Km**: Kilometers; **SD**: Standard Desvition

### Effects of Triathlon races on pro-inflammatory cytokine levels

All included studies evaluated the production of pro-inflammatory cytokines in response to different Triathlon races (CRP, IL-1, IL-1β, IL-1ra, IL-2, IL-6, IL-8, IL-12p40, IL-17, IL-23, INF-γ and TNF-α) [22–31] (Table 3**)**. Top-level Triathlon showed a significant increase of IL-8 and MCP-1 [22]. However, in the Olympic Triathlon race, there was an increase in levels of IL-1, IL-2, and TNF-α . The percentage of PBMCs secreting the pro-inflammatory cytokine IFN decreased after the Olympic Triathlon race [23]. Sprint Triathlon has increased levels of IL-6, IL-8, IFN−γ, and TNF-α [25]. Similarly, the Ironman Triathlon also increased TNF-α, as well as IL-1ra, IL-6, and IL-12p40 serum levels [31]. However, there was no significant difference in levels of IL-1β after the Ironman Triathlon race [31]. The Long-distance Triathlon promoted a significant decrease in the serum levels of IL-8 [24]. In contrast, there were no significant differences in the serum levels of IL-6 and TNF-α after Long-distance Triathlon [29]. In the World Championship race, there was a significant reduction of plasma levels of IL-1ra, IL-6, and IL-8 [30]. Finally, in the Ultraman Triathlon, there were no significant differences in the plasma levels of IL-6, IL-17, and IL-23 [31].

**Table 3:** Pro/anti-inflammatory cytokines production and metabolic markers in triathletes

Author, Year	Category Analysis	Cytokines	Other Metabolic Outcomes
Banfi, 2008	Top-level Triathlon Serum	↑ IL-8; MCP-1	↑ EGF; VEGF
Bassit, 2000	Olympic Triathlon PMBC	↑ IL-1; IL-2; TNF-α / ↓ INF-γ	˗
Huang, 2019	Sprint Triathlon Serum	↑ IL-4; IL-6; IL-8; IL-10; INF-γ; TNF-α	= CK; ↑ FFA; LDH; Lactate; Myostatin
Jenkendrup, 2008	Ironman Triathlon Serum	= TNF-α; IL-6	↑ CRP
Merino, 2006	Long-distance Triathlon Serum	↑ IL-1ra; IL-6; IL-10; ↓ IL-8	↑ CK; FFA; = CRP
Neubauer, 2008	Ironman Triathlon Serum	↑ IL-6; IL-10	↑ CK; CRP; Cortisol; Myostatin
Nieman, 2004	World Championship Serum	↑IL-6; IL-8; IL-1ra; IL-10	↑ Cortisol
Pinho, 2010	Ironman Triathlon Serum/Plasma	↑ IL-1ra; IL-6; IL-10; TNF-α	↑ CK
Smith, 2019	Ultraman Triathlon Plasma	= IL-6; IL-17; IL-23; IL-10	↑ CRP
Suzuki, 2006	Ironman Triathlon Plasma	↑ IL-1ra; IL-6; IL-10; IL-12p40; = IL-1β; IL-4	↑ CK; FFA; LDH

**Caption: CK**: Creatine Kinase; **CRP**: C-Reactive Protein; **EGF**: Endothelial Growth Factor; **FFA**: Free Fatty Acids; **IL-1**: Interleukin-1; **IL-1β**: Interleukin-1 Beta, **IL-1ra**: Interleukin-1 receptor agonist; **IL-2**: Interleukin-2; **IL-4**: Interleukin-4; **IL-6**: Interleukin-6; **IL-8**: Interleukin-8; **IL-10**: Interleukin-10; **IL-17**: Interleukin-17; **IL-23**: Interleukin-23; **IFN-γ**: Interferon-gamma; **LDH**: Lactate dehydrogenase; **MCP-1**: Monocyte Chemoattractant Protein-1;**PMBC**: Peripheral Mononuclear Blood Cells; **TNF-α**: Tumor Necrosis Factor-alpha; **VEGF**: Vascular Endothelial Growth Factor

### Triathlon races on anti-inflammatory cytokine levels

Seven studies evaluated the circulating levels of anti-inflammatory cytokines (IL-4 and IL-10) in response to the Triathlon race [24–28, 30, 31]. Ironman and Sprint Triathlon races were able to increase the levels of IL-4 and IL-10. Similarly, Long-Distance Triathlon increased IL-10 in serum and plasma levels [24, 26–28]. World Championship Triathlon demonstrated a significant decrease in the plasma of IL-10 [30]. No significant differences were found in plasma levels of IL-10 after the Ultraman triathlon [31].

### Triathlon races impact on metabolic markers

Nine of the studies included in this systematic review evaluated different metabolic markers linked to blood vessel growth and development (EGF and VEGF), muscle damage (CK, LDH, and Myostatin), muscle fatigue (FFA and Lactate), physiological stress (Cortisol) and acute inflammation phase (CRP) were evaluated in response to different Triathlon races [22, 24–31]. Only one study observed a significant increase in EGF and VEGF levels after Top-Level Triathlon [22]. Markers associated with muscle damage, we observed in four studies that after Long-Distance and Iron man Triathlon there was an increase in CK [24, 26–28]. Sprint Triathlon did not find differences CK levels [25]. Sprint and Ironman Triathlon races were able to significantly increase LDH and myostatin levels at serum and plasma levels [25, 26] and increased myostatin.

Long-Distance, Sprint, and Ironman Triathlon were able to increase systemic FFA levels, indicators of muscle fatigue [24, 25, 28]. High lactate levels were found in the Sprint Triathlon races [25]. Ironman and World Championship Triathlon race promoted an increase in cortisol [26, 30]. Finally, four studies evaluated Triathlon responses on CRP levels markers of acute inflammation. Long-distance, Iron, and Ultraman Triathlon events were able to significantly increase CRP levels [26, 29, 31]. On the other hand, one study did not observe significant differences in the levels of this protein after Long-Distance Triathlon [24].

### Methodological quality of studies

Table 4 summarizes the quality of the studies included. All 10 articles were rated as having a reasonable quality score (50–79%). Most studies presented the inclusion criteria, such as gender, age, and questionnaire filling, and all presented the context of the studies. The report was reliably evaluated with valid instruments and trained evaluators, furthermore, the objectives are in accordance with the methodological framework. Most studies did not present if they used strategies to identify and eliminate confounding variables.

**Table 4:** Study quality assessment - Joanna Briggs Institute

Studies	Q1	Q2	Q3	Q4	Q5	Q6	Q7	Q8	Score (%)
Banfi, 2008	Y	Y	Y	Y	N	N	Y	Y	75
Bassit, 2000	Y	Y	Y	Y	N	N	Y	Y	75
Huang, 2019	Y	Y	Y	Y	N	N	Y	Y	75
Jenkendrup, 2008	Y	Y	Y	Y	N	N	Y	Y	75
Merino, 2006	Y	Y	Y	Y	N	N	Y	Y	75
Neubauer, 2008	Y	Y	Y	Y	N	N	Y	Y	75
Nieman, 2004	Y	Y	Y	Y	N	N	Y	Y	75
Pinho, 2010	Y	Y	Y	Y	N	N	Y	Y	75
Smith, 2019	Y	Y	Y	Y	N	N	Y	Y	75
Suzuki, 2006	Y	Y	Y	Y	N	N	Y	Y	75
Banfi, 2008	Y	Y	Y	Y	N	N	Y	Y	75

**Caption**: Y - YES, N - No, U - Not clear. Q1: Was the inclusion criteria well defined? Q2: Have participants and context been described in detail? Q3: Were the measurements collected in a valid and reliable way? Q4: Were

standardized and objective inclusion criteria used? Q5 Were any confounding variables found? Q6: Were strategies used to deal with confounding variables? Q7: Were the results measured validly and reliably? Q8: Was the

statistical analysis used adequate?. The score for each article was calculated as a percentage and the quality of each study was classified as high (80–100%), fair (50–79%), or low (50%).

## Discussion

In the present systematic review, we aimed to evaluate the impacts of different triathlon events on the profile of cytokines (pro and anti-inflammatory) and metabolic markers in triathletes. First, we verified the increase of pro-inflammatory cytokines including IL-1, IL-2, IL-6, IL-8, IL-12p40, INF-γ, MCP-1, TNF-α at PBMC, serum, and plasma levels after different races of triathlon. Second, we observed an increase in the production of anti-inflammatory cytokines (IL-4 and IL-10) in most of the studies included after the Sprint, Long-Distance, and Ironman races at serum and plasma levels. Third, in the metabolic factors, we observed an increase in the concentration of markers in the blood and plasma of muscle damage (CK, LDH, and Myostatin), muscle fatigue (FFA and Lactate), physiological stress (Cortisol), and inflammation phase (CRP) in athletes of different events of triathlon.

Cytokines are signaling proteins produced by immune and non-immune cells that have cell signaling functions, positive and/or negative regulation of several genes and their transcription factors, and even stimulate or detain inflammation promoted by different stimuli including bacteria and viruses [32, 33]. Alves et al. 2022, demonstrated through a systematic review with meta-analysis that exposure to high running volume (exertion time, duration, and distance covered) is associated with a higher concentration of pro-inflammatory cytokines, including IL-1β, IL-8, and TNF-α. Furthermore, The serum levels of IL-1ra and IL-10 increased due to prolonged aerobic training [6]. However, the authors only considered the long-distance modalities (half marathon, marathon, and ultramarathon) excluding triathlon. Similarly, the data included in the present systematic observed that different triathlon races promoted an increase in PMBC, serum, or plasma concentration of pro-inflammatory cytokines [6, 9].

High concentrations of pro-inflammatory cytokines are observed at the end of triathlon races and can be explained by the volume of the race, including the intensity of the exercise. In contrast, they did not have an association with the triathlon distance. This result corroborates studies evaluating endurance athletes. Studies have observed leucocytosis and high serum levels of proinflammatory cytokines after marathon races [34–36]. The metabolic activity and damage observed in muscle cells as a result of long-distance races, such as triathlon, seem to serve as significant catalysts for the migration of some leucocytes, along with the release of cytokines. In addition, there are neuroendocrinological and metabolic multifactorial mechanisms involving extreme stimulus and underlying consequences. Strenuous physical exercise such as triathlon increases immunosuppression [9, 37, 38]. The possible relationship between physical exercise and UTRI can be explained and modeled by a “J” curve, which can occur both in competitions and training, usually caused by rhinovirus, adenovirus, and para-influenza virus [19, 39, 40]. In addition, this profile of disease involvement can impair health and performance-related physical fitness components such as maximal oxygen volume, respiratory coefficient, and lactate threshold [35, 36].

The anti-inflammatory response was evaluated by the serum levels of IL10 and IL4. Studies showed that strenuous physical exercise can increase IL-10 levels, being able to return to its basal level in the rest period [41]. Moreover, Santos et al. (2019) have shown that the magnitude of the plasma IL-10 increases is associated with exercise duration [42]. In addition, evidence has been indicated that the increase in IL-10 serum levels is correlated with low levels of chronic low-grade inflammation and tissue health [7]. Huang et al. 2019 have found an increase in plasma IL-4. Nevertheless, Suzuki et al., 2006 did not see any difference between pre and post-IL-4 serum levels. According to our findings, there is no significant IL-4 enhancement because of different protocols of Aerobic exercise [43]. Moreover, low IL-4 serum levels observed at the end of triathlon races can be explained by the strong inhibitory effects of IL-10, and IL-6 observed after long-distance triathlon races. These collectively contribute to averting excessive systemic inflammation [44].

Prolonged exercise protocols such as triathlons are known to cause changes in other biomarkers (gene expression and protein levels) [40]. It was spotted significant expansion of EGF and VEGF levels in many hematopoietic, endothelial cells, and smooth muscle cells of the vasculature into epithelial [40]. Moreover, evidence has shown that aerobic exercise must trigger the EGF and VEGF production and release due to physiologic adaptation to exercise, such as angiogenesis, indicating that EGF and VEGF are important biomarkers of aerobic exercise [45]. In parallel, the studies have noticed that CK plasma levels increased post-race. As observed in a randomized double-blind crossover study by Galan et al. 2018, the CK serum levels enhanced after treadmill running until exhaustion [46].

In addition, Danielsson et al., 2017 have related an increase in CK levels after an Ironman-distance triathlon, which has been associated with being male [8]. Next, it was known for an enhancement of FFA and LDH levels in Sprint, Ironman, and long-distance triathlons. Finally, the cortisol levels were grown during triathlon protocols. It is known that the physiological demands of long-distance running, such as triathlon, should cause an increase in FFA, LDH, cortisol, and lactate levels due to adaptation to the extensive energy expenditure of long-distance exercise protocols [47–49]. Finally, an increased level of Myostatin was stated in the aftermath of the Sprint and Iron Man triathlon according to previous evidence. Ben-Zaken et al., 2017 have obtained that Myostatin expression was linked with a favorable outcome in long-distance running performance [50].

Since chronic systemic inflammation can be considered a factor that influences the performance of triathlon athletes, recommendations for controlling the pillars of improving physical capacity (availability of nutrients, sleep behavior, strength training) are important to modulate the immune response. Furthermore, it reduces both physical and physiological distress while expediting the recovery and rehabilitation process from injuries. In this regard, Individuals who practice triathlons might benefit from the immunomodulatory effects of a strength exercise strategy combined with training for sport [51, 52]. In addition, adequate nutrient availability is known to benefit the immune function, including cell-mediated immunity and balanced inflammatory response. Finally, studies have shown that good sleep behavior could be a complementary approach to decreasing chronic inflammation [53, 54].

### Strengths and limitations

The present systematic review presents important limitations that should be considered in the generalization of the findings. Firstly, we considered different distances of the triathlon race, which means that the generalization of the findings should be specific. The limitations of this systematic review mostly involved the methods of the studies. For example, the lack of control for the covariates (such as age, nutrition state, sleep quality, etc.) can be an important source of bias among the studies included. Another important point is the characteristics of the sample included. As we only described the sex, and distance of participation, additional information, such as competitive level, training characteristics can be useful in future research.

Therefore, the heterogeneity in the quality of reference sources is the strength of this review, since it observed the efforts of the inflammatory cytokine’s serum levels, as well as biomarkers related to the performance in different triathlon races. On the other hand, it must be highlighted that the studies did not randomize their populations, a procedure recognized by PRISMA. Some studies did not investigate all the outcomes considered relevant in this scenario. However, we hold our work as relevant as it systematically summarizes the available evidence for future research to consider.

## Conclusions

Ultimately, this is the first systematic review that identified the impact of different triathlon tests on the pro- and anti-inflammatory cytokines and other metabolic molecules related to athletic performance. Thus, different triathlon tests have implied a release of pro and anti-inflammatory cytokines, some chemokines, and other metabolic markers associated with performance. Despite, this phenomenon does not lead to inflammatory exacerbations, it might also confer beneficial impacts on athletic performance, including increased strength production and muscular hypertrophy, as well as the development of cardiorespiratory capacity. Such processes can be explained due to the great metabolic capacity of skeletal muscle, which in response to different exercise and physical training protocols is capable of producing chemical mediators and transcriptional factors such as IL-6, Phosphatidylinositol 3-kinase (PI3K) and mechanistic target of rapamycin kinase (MTOR), which are part of the signaling of these biological processes, essential for growth, cellular and organic development. In addition, these significant physiological changes must return to standard levels during recovery time. However, more studies need to be done to investigate the magnitude of the effects of the different triathlon protocols on the immune system overall.

In practical considerations, the impact of triathlon on inflammation and metabolic profiles emerges as a critical determinant of performance. Healthy triathlon individuals should pay attention to factors such as nutrition, sleep behavior, and complementary training to reduce the degree of inflammation at the end of training and facilitate faster recovery.

## Data Availability

The data sets used in this systematic review are available in electronic databases and are the responsibility of each study author and the correspondent of this article.

## References

[1] Strock GA, Cottrell ER, Lohman JM (2006). Triathlon Phys Med Rehabil Clin N Am.

[2] Etxebarria N (2021). Running your best triathlon race. Int J Sports Physiol Perform.

[3] Knechtle B (2015). What predicts performance in ultra-triathlon races? - a comparison between Ironman distance triathlon and ultra-triathlon. Open Access J Sports Med.

[4] Arnold MJ, Moody AL (2018). Common running injuries: evaluation and management. Am Fam Physician.

[5] Bosu O (2016). Stretching for Prevention of Exercise-Related Injury. Am Fam Physician.

[6] Alves MDJ (2022). Changes in Cytokines Concentration following Long-Distance running: a systematic review and Meta-analysis. Front Physiol.

[7] Cerqueira É (2019). Inflammatory effects of High and Moderate Intensity Exercise-A systematic review. Front Physiol.

[8] Danielsson T (2017). Blood biomarkers in male and female participants after an ironman-distance triathlon. PLoS ONE.

[9] Domin R et al. *Effect of various Exercise regimens on selected Exercise-Induced cytokines in Healthy people*. Int J Environ Res Public Health, 2021. 18(3). 10.3390/ijerph18031261 PMC7908590 33572495

[10] Bentley DJ, Bishop D (2008). Science and medicine of triathlon. J Sci Med Sport.

[11] Vleck V, Millet GP, Alves FB (2014). The impact of triathlon training and racing on athletes’ general health. Sports Med.

[12] Maughan RJ, Fallah J, Coyle EF (2010). The effects of fasting on metabolism and performance. Br J Sports Med.

[13] Melin AK (2019). Energy Availability in Athletics: Health, Performance, and Physique. Int J Sport Nutr Exerc Metab.

[14] Ramezani Ahmadi A (2019). The effect of glutamine supplementation on athletic performance, body composition, and immune function: a systematic review and a meta-analysis of clinical trials. Clin Nutr.

[15] Tjoe JA (2020). Team triathlon effects on physiological, psychological, and immunological measures in women Breast cancer survivors. Support Care Cancer.

[16] Filgueira TO (2021). The relevance of a physical active lifestyle and physical fitness on Immune Defense: Mitigating Disease Burden, with Focus on COVID-19 consequences. Front Immunol.

[17] Middelbeek RJW (2021). Exercise intensity regulates cytokine and klotho responses in men. Nutr Diabetes.

[18] Atias-Varon D, Heled Y. *[STRENUOUS AND PROLONGED EXERCISE AND UPPER RESPIRATORY TRACT INFECTION - TREATMENT OR THREAT?]* Harefuah, 2017. 156(11): p. 730–734. 29198093

[19] Simpson RJ (2020). Can exercise affect immune function to increase susceptibility to Infection?. Exerc Immunol Rev.

[20] Moher D (2015). Preferred reporting items for systematic review and meta-analysis protocols (PRISMA-P) 2015 statement. Syst Rev.

[21] Munn Z (2019). The development of software to support multiple systematic review types: the Joanna Briggs Institute System for the Unified Management, Assessment and Review of Information (JBI SUMARI). Int J Evid Based Healthc.

[22] Banfi G (2008). Strenuous exercise activates growth factors and chemokines over-expression in human serum of top-level triathlon athletes during a competitive season. Clin Chem Lab Med.

[23] Bassit RA (2000). The effect of BCAA supplementation upon the immune response of triathletes. Med Sci Sports Exerc.

[24] Gomez-Merino D (2006). Comparison of systemic cytokine responses after a long distance triathlon and a 100-km run: relationship to metabolic and inflammatory processes. Eur Cytokine Netw.

[25] Huang WC et al. *The Beneficial effects of Lactobacillus plantarum PS128 on High-Intensity, Exercise-Induced oxidative stress, inflammation, and performance in triathletes*. Nutrients, 2019. 11(2). 10.3390/nu11020353 PMC6412901 30736479

[26] Neubauer O, König D, Wagner KH (2008). Recovery after an Ironman triathlon: sustained inflammatory responses and muscular stress. Eur J Appl Physiol.

[27] Pinho RA (2010). Oxidative stress and inflammatory parameters after an Ironman race. Clin J Sport Med.

[28] Suzuki K (2006). Changes in markers of muscle damage, inflammation and HSP70 after an Ironman Triathlon race. Eur J Appl Physiol.

[29] Jeukendrup AE (2000). Relationship between gastro-intestinal complaints and endotoxaemia, cytokine release and the acute-phase reaction during and after a long-distance triathlon in highly trained men. Clin Sci (Lond).

[30] Nieman DC (2004). Vitamin E and immunity after the Kona Triathlon World Championship. Med Sci Sports Exerc.

[31] Smith KA (2020). Ultra-endurance triathlon performance and markers of whole-body and gut-specific inflammation. Eur J Appl Physiol.

[32] Borish LC, Steinke JW (2003). 2. Cytokines and chemokines. J Allergy Clin Immunol.

[33] Opal SM, DePalo VA (2000). Anti-inflammatory Cytokines Chest.

[34] Santos VC (2016). Marathon Race affects Neutrophil Surface molecules: Role of Inflammatory mediators. PLoS ONE.

[35] Grabs V (2017). Rutoside and Hydrolytic enzymes do not attenuate Marathon-Induced inflammation. Med Sci Sports Exerc.

[36] Passos BN et al. *Association of Daily Dietary Intake and Inflammation Induced by Marathon Race* Mediators Inflamm, 2019. 2019: p. 1537274. 10.1155/2019/1537274 PMC6800895 31686980

[37] Gleeson M. *Immune function in sport and exercise* J Appl Physiol (1985), 2007. 103(2): p. 693-9. 10.1152/japplphysiol.00008.2007 17303714

[38] Gokhale R, Chandrashekara S, Vasanthakumar KC (2007). Cytokine response to strenuous exercise in athletes and non-athletes–an adaptive response. Cytokine.

[39] O’Keefe EL (2020). Training for longevity: the reverse J-Curve for Exercise. Mo Med.

[40] Nieman DC (1994). Exercise, Infection, and immunity. Int J Sports Med.

[41] Moldoveanu AI, Shephard RJ, Shek PN (2001). The cytokine response to physical activity and training. Sports Med.

[42] Cabral-Santos C (2019). Interleukin-10 responses from acute exercise in healthy subjects: a systematic review. J Cell Physiol.

[43] Zheng G (2019). Effect of Aerobic Exercise on inflammatory markers in healthy middle-aged and older adults: a systematic review and Meta-analysis of Randomized controlled trials. Front Aging Neurosci.

[44] Leal LG, Lopes MA, Batista ML (2018). Physical Exercise-Induced myokines and muscle-adipose tissue crosstalk: a review of current knowledge and the implications for Health and Metabolic Diseases. Front Physiol.

[45] Timmons JA (2005). Modulation of extracellular matrix genes reflects the magnitude of physiological adaptation to aerobic exercise training in humans. BMC Biol.

[46] Galan BS (2018). Effects of taurine on markers of muscle damage, inflammatory response and physical performance in triathletes. J Sports Med Phys Fitness.

[47] Buonocore D (2020). Effect of 8-week n-3 fatty-acid supplementation on oxidative stress and inflammation in middle- and long-distance running athletes: a pilot study. J Int Soc Sports Nutr.

[48] Hausswirth C, Lehénaff D (2001). Physiological demands of running during long distance runs and triathlons. Sports Med.

[49] Nagel D, Seiler D, Franz H (1992). Biochemical, hematological and endocrinological parameters during repeated intense short-term running in comparison to ultra-long-distance running. Int J Sports Med.

[50] Ben-Zaken S (2017). The combined frequency of IGF and myostatin polymorphism among track & field athletes and swimmers. Growth Horm IGF Res.

[51] Ziegler AK (2019). The effect of resistance exercise upon age-related systemic and local skeletal muscle inflammation. Exp Gerontol.

[52] Rall LC (1996). Effects of Progressive resistance training on immune response in aging and chronic inflammation. Med Sci Sports Exerc.

[53] Hepsomali P, Groeger JA (2022). Examining the role of systemic chronic inflammation in diet and sleep relationship. J Psychopharmacol.

[54] Lee WJ (2017). Long sleep duration, Independent of frailty and chronic inflammation, was associated with higher mortality: a national population-based study. Geriatr Gerontol Int.

